# Identifying Individual Risk Factors and Documenting the Pattern of Heat-Related Illness through Analyses of Hospitalization and Patterns of Household Cooling

**DOI:** 10.1371/journal.pone.0118958

**Published:** 2015-03-05

**Authors:** Michael T. Schmeltz, Grace Sembajwe, Peter J. Marcotullio, Jean A. Grassman, David U. Himmelstein, Stephanie Woolhandler

**Affiliations:** 1 School of Public Health, City University of New York (CUNY), New York, United States of America; 2 Hunter College, City University of New York (CUNY), New York, United States of America; 3 CUNY Institute for Sustainable Cities, New York, United States of America; 4 Health and Nutrition Sciences, Brooklyn College, City University of New York (CUNY), Brooklyn, NY, United States of America; NASA Marshall Space Flight Center, UNITED STATES

## Abstract

**Background:**

As climate change increases the frequency and intensity of extreme heat events researchers and public health officials must work towards understanding the causes and outcomes of heat-related morbidity and mortality. While there have been many studies on both heat-related illness (HRI), there are fewer on heat-related morbidity than on heat-related mortality.

**Objective:**

To identify individual and environmental risk factors for hospitalizations and document patterns of household cooling.

**Methods:**

We performed a pooled cross-sectional analysis of secondary U.S. data, the Nationwide Inpatient Sample. Risk ratios were calculated from multivariable models to identify risk factors for hospitalizations. Hierarchical modeling was also employed to identify relationships between individual and hospital level predictors of hospitalizations. Patterns of air conditioning use were analyzed among the vulnerable populations identified.

**Results:**

Hospitalizations due to HRI increased over the study period compared to all other hospitalizations. Populations at elevated risk for HRI hospitalization were blacks, males and all age groups above the age of 40. Those living in zip-codes in the lowest income quartile and the uninsured were also at an increased risk. Hospitalizations for HRI in rural and small urban clusters were elevated, compared to urban areas.

**Conclusions:**

Risk factors for HRI include age greater than 40, male gender and hospitalization in rural areas or small urban clusters. Our analysis also revealed an increasing pattern of HRI hospitalizations over time and decreased association between common comorbidities and heat illnesses which may be indicative of underreporting.

## Introduction

Heat from high environmental temperatures is a natural hazard that can adversely affect human health [1–3]. Extreme heat events account for more fatalities in the United States than any other weather hazard, with approximately 650 deaths per year [4]. As climate change increases the frequency and intensity of extreme heat events researchers and public health officials must work aggressively towards understanding the causes and outcomes of heat-related morbidity and mortality [5–7]. While there have been many studies on both heat-related morbidity and mortality [8–10], there are fewer studies on heat-related morbidity than those which focus on heat-related mortality [11–15]. The effect of temperature on morbidity is a significant public health concern that will escalate as global mean temperature continues to increase and more episodes of extreme heat events affect large geographic regions [16].

Human populations have acclimatized to a variety of local climates physiologically and behaviorally, though there are limits to the extremes in temperature the human body can tolerate. An individual’s ability to thermoregulate protects the body from ambient temperatures by maintaining a core body temperature around 36.0°C to 37.5°C [17]. Body temperatures outside of this range are classified as either hypothermia (body temperature below 35.0°C) or hyperthermia (body temperature above 37.5°C) [18,19]. Heat-related illnesses (HRIs) are a spectrum of conditions ranging from minor to life threatening. They include heat stress, heat exhaustion and heat stroke. Heat stress is a perceived discomfort and physiological strain; heat exhaustion, a mild to moderate illness; and heat stroke, a severe illness characterized by a core body temperature above 40.0°C [19]. Clinically, severe heat illness can be seen as a combination of systematic inflammatory response and cytotoxicity, which left untreated results in multiple organ failure [17,19,20]. Infants are at increased risk of heat-related illness as are the elderly, individuals taking medications that interfere with thermoregulation, and those with underlying medical conditions such as cardiac, respiratory, renal disease or diabetes [3,21,22].

Studies of heat mortality during specific events, such as the 1995 Chicago heat wave and the 2003 heat wave that affected much of Europe, highlight characteristics of populations that are at risk of dying during extreme heat events [9,23]. These studies described elevated heat mortality among the elderly, persons of color or of lower socioeconomic status, individuals confined to bed or unable to care for themselves and individuals with social risk factors such as not leaving the home daily, living alone, and limited social networks [23,24]. Studies have also identified underlying medical conditions as risk factors for heat mortality, including cardiovascular, cerebrovascular, respiratory, renal and neurologic diseases, and diabetes [25,26]. However, the diagnosis of a specific heat-related illness is not always documented during heat events and diagnoses for cause of death are usually listed as the underlying medical condition [27–29]. Some studies of heat-related morbidity have focused on emergency dispatch data and emergency department visits during individual heat events, with specific HRI diagnoses [30,31], while others have analyzed excess hospitalizations during specific extreme heat events [13,26,32]. Few investigations have investigated HRI hospitalizations across different geographic regions, perhaps because classification of heat events often varies across regions [15,33]. Additionally the underreporting of HRI diagnoses may limit our understanding of health-related illness [34].

One of the strongest protectors against HRIs is ownership and use of an air conditioner [35–37]. As of 2009, 87% of homes in the United States have air conditioning, although the prevalence of air conditioning is lower (and less efficient cooling systems such as fans, are higher) among apartment dwellers and low income households [38]. A recent Morbidity and Mortality Weekly Report highlighting heat illness and deaths in New York City indicated that a majority of heat illness decedents did not have a working air conditioner, although information on cooling practices of decedents was limited [39]. Research is needed regarding a number of economic and behavioral issues that might improve access to air conditioning and reduce heat mortality and morbidity [40,41].

In addition to individual level determinants and behaviors around cooling during extreme heat events, the environment plays a key role in exacerbating or protecting us from extreme heat events. While the urban heat island (UHI) is not a new concept, it has been used to explain higher rates of HRI in urban areas [42,43]. The average ambient urban temperature is increased where vegetation has been replaced by heat-retaining materials such as asphalt and concrete. Recent studies using remote sensing and spatial analysis attempt to create heat vulnerability maps and indexes based on environmental factors [44–49]. An integrated understanding of environmental factors, individual-level factors and socio-behavioral determinants is needed to accurately identify populations most vulnerable to extreme events due to climate change. Health impacts associated with heat waves disproportionately harm vulnerable populations. However, with the increase in frequency and magnitude of climate related disasters, extreme heat events may ultimately disrupt and affect all populations. This research examines individual and environmental risk factors for HRI hospitalization using a large nationally-representative administrative database. In addition we document patterns of household cooling, with particular focus on populations at risk for HRI hospitalization.

## Materials and Methods

Our primary source of data is the 2001 to 2010 Healthcare Cost and Utilization Project (HCUP) Nationwide Inpatient Sample (NIS), which was developed by the Agency for Healthcare Research and Quality (AHRQ). The NIS is an all-payer database (i.e. contains patients without regard to their health insurance), with data from approximately 8 million hospitalizations per year (about 20% of all hospital discharges in the United States) [50]. The AHRQ provides a weighting variable for descriptive and regression analyses to provide a better estimate of error and statistical significance for national estimates. The NIS includes data on patient demographics and hospital characteristics, primary and secondary diagnoses coded according to the *International Classification of Diseases*, *Ninth Revision* (ICD-9) (with up to 15 diagnoses available for the years 2001–2008 and up to 25 diagnoses for 2009–2010), primary and secondary procedures (ICD-9 coding up to 15 diagnoses available for the years 2001–2008 and up to 25 diagnoses for 2009–2010), type and source of admission, discharge disposition, primary payer type, total hospital charges, and length of stay. Stratification and weighting variables allow calculation of national estimates that account for the complex sampling design [50,51]. These data include information on urban vs. rural hospital location. Additional spatial data for geocoded hospital location was obtained from ESRI based on American Hospital Association (AHA) identifiers, and merged to the NIS dataset to create a detailed and updated hospital location indicator. Using the 2010 Census Urban and Rural classification, geocoded hospitals were mapped and designated as being in urbanized areas of 50,000 or more people, in small urban clusters of 2,500 to 50,000 people or in rural areas which encompassed all populations outside urbanized areas and small urban clusters [52]. All spatial joinings were done using ArcGIS (version 10; ESRI, Redlands, CA).

Data on air conditioner use was derived from the Residential Energy Consumption Survey (RECS), a national household survey conducted by the United States Energy Information Administration (EIA) starting in 1978. In 2005 and 2009 surveys were collected from approximately 4,000 and 12,000 households, respectively, which were nationally representative of the 110 million residential U.S. households. We analyzed RECS data on air conditioner ownership and use examining households below 100% of the federal poverty line, and below 150% of the federal poverty line, employment status, and age of householder (head of household) [38].

The study population for HRI consists of patients in the 2001–2010 NIS with at least one diagnosis of a heat-related illness (ICD-9 codes 992.0–992.9). The control group consisted of all other NIS hospitalizations during this time period. The HRI variable was coded as a binary indicator variable. The initial cohort consisted of 15,885 patient records for heat-related illness (HRI) and 79,140,110 patient records for controls. Since approximately 95% of all HRI hospitalizations occurred in the summer months (May-September), hospitalizations in other months were excluded yielding a final cohort of 14,949 HRI patient records and 37,019,792 controls.

The outcome variable was heat-related illness. First, a descriptive bivariate analysis of baseline characteristics was performed for HRI and control populations. Chi-square tests and t-tests were used to examine differences between these populations. Second, multivariable analysis was performed to determine risk factors associated with heat morbidity. A log-binomial model, which directly models risk ratios (RRs), was used. Analyses were weighted for national estimates and to enhance likelihood ratios [53]. Risk factors for heat-related illnesses were explored, including patient characteristics (gender, age, race, zip-code income quartile, insurance, comorbidities), and hospital characteristics (hospital size—based on number of beds, hospital location, hospital region). Patient comorbidities were analyzed using the AHRQ algorithms based on methods developed by Elixhauser et al [54]. Third, a multilevel (i.e. hierarchical) model was used to better account for the correlation between patient and hospital-level characteristics. Weighting was not used for multilevel analysis as recommended by the HCUP hierarchical modeling report [55]. Lastly air conditioner data was used to analyze the ownership and use of air conditioning among vulnerable populations as identified in the hospitalization data.

All statistical analyses were performed using SAS 9.3. All data used in this study are publically available and had been anonymized and de-identified prior to our analyses. The study was a secondary data analysis and data were de-identified prior to being obtained by the authors and did not require patient consent. This study was also approved by the Institutional Review Board of Hunter College, CUNY and conforms to the HCUP data use agreement for data protection.

## Results

**Fig 1:**
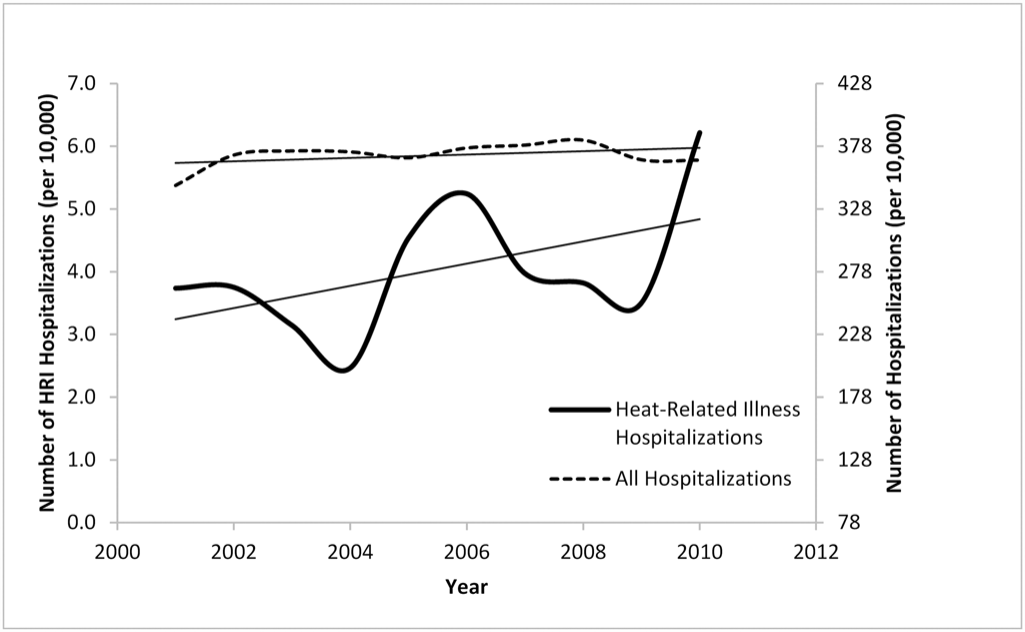
Heat-Related Illness versus All Hospitalizations Rates, Summer 2001–2010. The dotted line represents the rate of all hospitalizations during the study time period which remained approximately level over time. The solid line represents heat-related illness hospitalizations which although varied by year have steadily increased over the study period.

The characteristics of patients with HRI and of all hospitalized patients during the five “summer” months from 2001 to 2010 in the NIS data are shown in Tables 1 and 2. After weighting there were 73,185 patient discharges for HRI and 181,094,795 for all other discharges during this time period. Compared to all non-HRI hospitalizations, more HRI admissions were emergencies. Emergency department (ED) admission (as source of admission) accounted for 59% for HRI hospitalizations and 34% of non-HRI hospitalizations, while emergency admission (as type of admission) accounted for 73% for HRI and 41% of non-HRI hospitalizations. The mean age was also higher for HRI than non-HRI hospitalizations, 55 years compared to 48 years. These differences were statistically significant (*p* < 0.05). The number of HRI hospitalizations also increased faster than non-HRI hospitalizations, albeit with large year-to-year variation (Fig. 1).

**Table 1:** Demographic Characteristics of Heat-Related Illness (HRI) patients, 2001–2010 (Summer).

	HRI (Not weighted), N	HRI (Weighted), %	All Hospitalization (Weighted), %
**Total**	14,949	73,185 (100%)	181,094,795 (100%)
**Age, Mean(±SD)**	55 (21.62)	—	48 (27.9)
**Gender**			
Male	10,998	72.99%	41.33%
Female	3,937	26.91%	58.66%
Missing	14	0.1%	0.01%
**Age Group (years)**			
0–17	669	4.45%	16.56%
18–39	3,088	20.54%	21.60%
40–64	5,709	38.17%	27.56%
65–74	1,995	13.41%	13.02%
75+	3,488	23.43%	21.26%
**Race/Ethnicity**			
White	8,213	54.93%	52.30%
Black	2,089	13.92%	11.20%
Hispanic	1,406	9.35%	10.40%
Other	566	3.77%	4.81%
Missing	2,675	18.02%	21.29%
**Median Income Quartile** *			
0–25th percentile	4,458	29.92%	22.86%
26th to 50th percentile^#^	3,210	21.44%	20.87%
51st to 75th percentile	2,345	15.59%	18.68%
76th to 100th percentile	1,716	11.42%	16.13%
Missing	3,200	21.63%	21.45%
**Payment Source**			
Medicare	5,897	39.6%	36.99%
Medicaid	1,365	9.14%	20.83%
Private Insurance	4,161	27.79%	34.83%
Self-Pay/Other	3,476	23.14%	9.01%
Missing	50	0.33%	0.16%
**Insurance Status**			
Insured	11,423	76.52%	90.82%
Uninsured	2,261	15.05%	5.81%
Missing	1,265	8.43%	3.36%
**Source of Admission** †			
Emergency Department	8,879	58.89%	33.95%
Other Hospital/Facility	280	1.86%	3.51%
Court/Law	7	0.05%	0.09%
Routine/Other	1,719	11.51%	38.89%
Missing	4,064	27.7%	23.56%
**Type of Admission** ‡			
Emergency	10,870	72.76%	40.94%
Urgent	2,088	14.13%	17.28%
Elective	784	5.23%	22.18%
Other	22	0.15%	9.65%
Missing	1,185	7.74%	9.99%

* based on zip code of patient

^#^ Median zip-code income

†source of the admission (patients was admitted to the hospital from the emergency department, a physician's office, nursing home etc.)

‡type of admission (emergency, urgent, elective, other.)

**Table 2:** Hospital Characteristics of Heat-Related Illness (HRI) patients, 2001–2010 (Summer).

	HRI (Not weighted), N	HRI (Weighted), %	All Hospitalizations (Weighted), %
**Total**	14949	72811 (100%)	180042390 (100%)
**Hospital Region**			
Northeast	1733	11.61%	17.13%
Mid-West	2788	18.75%	19.98%
South	8120	54.43%	45.62%
West	2308	15.22%	17.27%
**Hospital Size (by # of beds)**			
Small	2553	17.15%	11.88%
Medium	3980	26.59%	24.45%
Large	8344	55.77%	63.34%
Missing	72	0.48%	0.32%
**Hospital Location** *			
Rural	574	3.81%	2.21%
Small Urban Cluster	1607	10.76%	6.37%
Urbanized Area	7255	48.72%	64.67%
Missing	5513	37.01%	26.75%

*Urbanized areas >50,000 people; Small Urban Cluster 2,500–50,000 people; Rural areas <2,500 people

Multivariable analyses identifying risk factors for patients with an HRI diagnosis and for any other hospitalization are shown in Table 3. Blacks had an elevated risk ratio of 1.21 (95% CI: 1.19–1.23) compared to Whites; males had a markedly higher risk ratio of 3.54 (95% CI: 3.49–3.58) compared to females; as did each age group above the age of 40 (ages 40–64) RR 1.34 (95% CI: 1.32–1.37), (ages 65–74) RR 1.28 (95% CI: 1.25–1.30), (ages 75+) RR 1.52 (95% CI: 1.47–1.55) compared to ages 18–39. Patients who lived in zip codes with the lowest median income quartile were more likely to be hospitalized for a HRI, RR 1.19 (95% CI: 1.17–1.21) as were those in the second lowest income quartile RR 1.08 (95% CI: 1.07–1.10) compared to the highest zip code income quartile. Lack of health insurance was a particularly strong social predictor of being hospitalized due to HRI, RR 2.33 (95% CI: 2.29–2.37).

**Table 3:** Multivariable model of risk factors of hospitalization for heat-related illnesses, United State 2001–2010 (Summer).

Variables	Unadjusted	Adjusted#	
	RR† (95%CI)††	RR (95%CI)	*p*-Value
**Race**			
White	1 *ref*.	1 *ref*.	
Black	1.18 (1.17, 1.20)	1.21 (1.19, 1.23)	<0.001
Hispanic	0.86 (0.84, 0.87)	0.88 (0.86, 0.90)	<0.001
Other	0.75 (0.73, 0.76)	0.88 (0.85, 0.90)	<0.001
**Gender**			
Female	1 *ref*.	1 *ref*.	
Male	3.13 (3.08, 3.18)	3.54 (3.49, 3.58)	<0.001
**Age Group (years)**			
0–17	0.28 (0.27, 0.29)	0.26 (0.25, 0.27)	<0.001
18–39	1 *ref*.	1 *ref*.	
40–64	1.45 (1.42, 1.47)	1.34 (1.32, 1.37)	<0.001
65–74	1.08 (1.06, 1.09)	1.28 (1.25, 1.30)	<0.001
75+	1.15 (1.14, 1.17)	1.52 (1.49, 1.55)	<0.001
**Median Income Quartile**			
0–25th percentile	1.85 (1.83, 1.87)	1.19 (1.17, 1.21)	<0.001
26th to 50th percentile*	1.46 (1.44, 1.47)	1.08 (1.07, 1.10)	<0.001
51st to 75th percentile	1.18 (1.17, 1.20)	0.97 (0.95, 0.98)	<0.001
76th to 100th percentile	1 *ref*.	1 *ref*.	
**Payment Source**			
Medicare	1.33 (1.29, 1.39)		
Medicaid	0.60 (0.57, 0.63)		
Private Insurance	1 *ref*.		
Self-Pay/Other	3.17 (3.13, 3.21)		
**Insurance Status**			
Insured	1 *ref*.		
Uninsured	3.09 (3.05, 3.11)	2.33 (2.29, 2.37)	<0.001
**Co-Morbidities**			
Congestive Heart Failure	0.80 (0.78, 0.81)	0.80 (0.78, 0.82)	<0.001
Chronic Pulmonary Disease	0.79 (0.78, 0.80)	0.72 (0.71, 0.82)	<0.001
Diabetes (no complications)	1.08 (1.06, 1.09)	0.90 (0.89, 0.92)	<0.001
Diabetes (complications)	0.75 (0.73, 0.78)		
Hypertension	1.30 (1.29, 1.31)	1.00 (0.99, 1.01)	0.8093
Fluid and Electrolyte Disorders**	7.76 (7.70, 7.81)		
Other Neurologic Disorders	1.98 (1.96, 2.00)	1.79 (1.75, 1.82)	<0.001
Obesity	0.79 (0.78, 0.80)		
Peripheral Vascular Disorders	0.66 (0.64, 0.68)		
Pulmonary Circulation Disorders	0.60 (0.57, 0.63)		
Renal Failure	0.77 (0.75, 0.78)	0.61 (0.60, 0.63)	<0.001
Psychoses	1.99 (1.96, 2.02)	1.83 (1.79, 1.87)	<0.001
**Hospital Region**			
Northeast	0.71 (0.70, 0.72)	0.72 (0.70, 0.73)	<0.001
Mid-West	1 *ref*.	1 *ref*.	
South	1.27 (1.26, 1.29)	0.94 (0.92, 0.95)	<0.001
West	0.94 (0.93, 0.95)	1.23 (1.20, 1.25)	<0.001
**Hospital Size (by # of beds)**			
Small	1.68 (1.66, 1.69)	1.39 (1.37, 1.42)	<0.001
Medium	1.24 (1.23, 1.25)	1.25 (1.23, 1.27)	<0.001
Large	1 *ref*.	1 *ref*.	
**Hospital Location** ^##^			
Rural	2.32 (2.28, 2.36)	1.79 (1.74, 1.83)	<0.001
Small Urban Cluster	2.27 (2.24, 2.29)	2.09 (2.06, 2.12)	<0.001
Urbanized Area	1 *ref*.		

† RR is Risk Ratio

†† P-values for unadjusted models were <0.001

*Median zip-code income

**We interpret this as part of the HRI rather than comorbidity per se.

^#^Adjusted for race, gender, age, median income zip code quartile, insurance status, comorbidities, hospital-bed size,-region, and-location.

^##^Urbanized areas >50,000 people; Small Urban Cluster 2,500–50,000 people; Rural areas <2,500 people

Patients with neurological disorders and psychoses were more likely to be hospitalized with HRI, with a relative risk of 1.79 (95% CI: 1.75–1.82) and 1.83 (95% CI: 1.79–1.87) respectively. Other comorbidities such as cardiovascular diseases, diabetes, renal failure and pulmonary diseases did not predict HRI relative to non-HRI hospitalizations with risk ratios of 0.80 (95% CI: 0.78–0.82) for congestive heart failure, 0.72 (95% CI: 0.71–0.82) for chronic pulmonary diseases, 0.90 (95% CI: 0.89–0.92) for diabetes, 1.00 (95% CI: 0.99–1.01) for hypertension and 0.61 (95% CI: 0.60–0.63) for renal failure. Examination of hospital characteristics indicate that patients in the Western U.S. region had higher risk of HRI hospitalization, RR 1.23 (95% CI: 1.20–1.25), compared to those in the Northeastern US, as did patients at small and medium sized hospitals (RR 1.39 95% CI: 1.37–1.42 and RR 1.25 95% CI: 1.23–1.27 respectively), compared to large hospitals. Hospitals located in rural areas, (RR 1.79 95% CI: 1.74–1.83) and in small urban clusters (RR 2.09 95% CI: 2.06–2.12) had increased risk of HRI hospitalizations as compared to those in urban areas.

The multilevel analysis (Table 4), found risk ratios similar to the single-level multivariable analysis: Blacks (RR 1.17, 95% CI: 1.08–1.27), males (RR 3.55, 95% CI: 3.35–3.77) and patients above the age of 40 (ages 40–64) (RR 1.29, 95% CI: 1.19–1.40), (ages 65–74) (RR 1.15, 95% CI: 1.04–1.27), (ages 75+) (RR 1.40, 95% CI: 1.28–1.53). Being in the lowest zip code income quartile significantly predicted of HRI at RR 1.13 (95% CI: 1.03–1.25) as did being uninsured (RR 2.51, 95% CI: 2.31–2.72). Hospital level characteristics for the multilevel analysis also had similar results with small hospital size having a relative risk of 1.35 (95% CI: 1.19–1.53) and medium hospital size a relative risk of 1.17(95% CI: 1.05–1.30). Risk of HRI hospitalization in rural areas and small urban clusters also remained higher than in urban areas. Analyses of the energy use data revealed that overall air conditioner use for all U.S. households increased from 82% in 2005 to 87% in 2009. However, households below 100% to 150% of the federal poverty line had 3% to 6% decrease in using an air conditioner from 2005 to 2009. Air conditioner use among householders who were unemployed, retired or employed part-time was 25% less likely in both 2005 and 2009, than among householders employed full-time. Additionally, air conditioner use increased among householders of most age groups from 2005 to 2009, but decreased by 13% among householders aged 75 and older.

**Table 4:** Multi-level multivariable model of risk factors of hospitalization for heat-related illnesses, United State 2001–2010 (Summer).

Variables			
	RR†	(95%CI)	p Value
**Race**			
White	1 *ref*.		
Black	1.17	1.08–1.27	0.002
Hispanic	0.93	0.84–1.03	0.148
Other	0.91	0.79–1.05	0.186
**Gender**			
Female	1 *ref*.		
Male	3.55	3.35–3.77	<0.001
**Age Group (years)**			
0–17	0.26	0.22–0.30	<0.001
18–39 (Ref)	1 *ref*.		
40–64	1.29	1.19–1.40	<0.001
65–74	1.15	1.04–1.27	0.007
75+	1.40	1.29–1.53	<0.001
**Median Income Quartile** *			
0–25th percentile	1.13	1.03–1.25	0.009
26th to 50th percentile (median)	1.05	0.96–1.15	0.258
51st to 75th percentile	0.97	0.89–1.06	0.463
76th to 100th percentile	1 *ref*.		
**Insurance Status**			
Insured	1 *ref*.		
Uninsured	2.51	2.31–2.72	<0.001
**Hospital Region**			
Northeast	0.72	0.61–0.86	0.002
Mid-West	1 *ref*.		
South	1.13	0.96–1.32	0.160
West	1.12	0.94–1.32	0.213
**Hospital Size (by # of beds)**			
Small	1.35	1.19–1.53	<0.001
Medium	1.17	1.05–1.30	0.004
Large	1 *ref*.	0.77–0.95	
**Hospital Location** **			
Rural	1.93	1.57–2.37	<0.001
Small Urban Cluster	2.00	1.75–2.29	<0.001
Urbanized Area	1 *ref*.		

† RR is Risk Ration and adjusted for race, gender, age, median income zip code quartile, insurance, hospital-bed size,-region, and-location.

* Median zip-code income

** Urbanized areas >50,000 people; Small Urban Cluster 2,500–50,000 people; Rural areas <2,500 people

## Discussion

Most of the risk factors for heat related morbidity that we identified were similar to those found in previous heat-related morbidity and mortality studies [27,56,57]. For instance persons identified as Black, elderly and of lower socioeconomic status were at a higher risk for HRI hospitalizations. A recent study examining United States emergency department visits for acute heat illnesses indicated that males were at a higher risk, as did we [56].

However, our nationally representative data yielded some surprising findings as well. First, all older age groups were at an increased risk of heat-related illness compared to 18–39 year olds. Prior studies have frequently highlighted only infants and elderly as being most vulnerable, especially to heat mortality [58,59]. This increased risk of hospitalization among middle-aged adults, not just the elderly may signal a lack of risk perception among the population in regards to heat-related illness. Studies have shown that adults and many elderly populations do not perceive themselves as at risk for a HRI [60]. A study of older people in London and Norwich, United Kingdom found that while people are able to recognize warnings and provide examples of what to do to reduce the effects of heat, many did not apply these warnings or behaviors as they failed to consider themselves at risk [61].

Second, much research has been done on the urban heat island effect and its implications on heat-related morbidity and mortality [62]; our study indicates that heat morbidity is not restricted to urban environments. Risk of hospitalization due to HRI was actually higher in small urban clusters (populations 2,500 to 50,000) and rural areas as compared to urban areas (populations 50,000+). This is in contrast to current research indicating that the urban heat island is exacerbating warming in urban areas. A recent study from China indicated that summer mortality rates have increased in large urban areas like Shanghai compared to suburban locales [63]. Additional research is needed to understand why risk of HRI hospitalization is higher in small urban clusters and rural areas, despite the fact that urban areas have higher temperatures, and hold heat for longer periods of time. Hospital travel distance, absent heat-health warning systems and social factors such as higher rates of manual labor and lower health literacy outside of urban areas may explain these differences.

Third, surprisingly, we found that common serious comorbidities, aside from neurological disorders and psychoses, were not a significant risk factor for being hospitalized for a heat-related illness compared to all other hospitalizations. This is contrary to previous research indicating that these common comorbidities are exacerbated during extreme heat events [12,24]. We interpret this cautiously as it may indicate only that other hospitalizations (i.e. our comparator) are very frequent for persons with “chronic” medical conditions. Additionally artifacts may have been introduced if the severity of the underlying condition and comorbidities led providers to under-diagnose or under-code HRI. Such underreporting obstructs our understanding of risk factors for heat-related illnesses.

Lastly, our results also indicate that individuals from poorer neighborhoods and especially persons without health insurance are at an increased risk of hospitalizations due to heat-related illnesses. Although low socioeconomic status has been shown to be a predictor for heat morbidity our insurance status indicator measures a specific aspect of socioeconomic status among hospitalizations of heat-related illnesses.

As pervasive as air conditioning use has become in the United States, many among these vulnerable populations still lack access to this protective factor [64]. Our air conditioning data showed that poor and elderly householders, as well as those who are unemployed or underemployed have lower rates of air conditioning use. These same populations are at the highest risk of being hospitalized for heat-related illnesses. In some communities air conditioning is still considered a luxury, but the increase in extreme heat events may make air conditioning a necessity. Steps are needed to improve the availability and affordability of air conditioning and to improve access to cooling centers. One study from California (where temperature variation is less extreme than in other parts of the U.S) indicated that while central air conditioning had a protective effect on heat morality, central air conditioning use was not associated with income [36]. However, the study did not analyze the use of room air conditioning or use of cooling centers which may impact lower socioeconomic populations. While air conditioning may prevent heat-related illness hospitalizations, equitable policies surrounding energy consumption during summer months and during heat events will become an important topic as the demand for energy increases and further stresses energy distribution systems [65].

To our knowledge this is one of the few studies to assess HRI nationally [46,56]. However, it has limitations. Administrative data is less accurate than clinical data and HRI is almost certainly under-coded. Because the NIS does not capture information regarding hospital readmission, an individual can be counted multiple times if hospitalized repeatedly for HRI. We were unable to account for clustered patient data and thus may slightly underestimate the width of our confidence intervals, although given the large data set we believe it should make little difference in the overall results. The NIS is a 20% sample survey and may be subject to sampling error. A sub-population may seriously be over-represented or under-represented. To overcome this limitation in the sampling design we included the use of weights that allow for national estimates. Reporting for some hospital level variables also varied from state to state. Additionally, the RECS data does not link with NIS records; thus comparisons could only be made at an ecological level concerning air conditioning use.

Heat is the number one cause of death from natural disasters in the United States [4], yet is often overlooked due to the limited visibility of heat as a hazard. Tornados, hurricanes, floods and blizzards are more tangible and dramatic than heat waves. The latest Intergovernmental Panel on Climate Change (IPCC) states that global mean temperature is projected to increase between 1.5 and 2.3°C by mid-century and over 4°C by the end of the century [66]. As temperatures increase the intensity and frequency of extreme heat events will also increase. In turn this will likely increase the number of hospitalizations of heat-related illnesses. Our research is pertinent in understanding the predictors for HRI hospitalizations and identifies three key risk factors for heat morbidity, including ages greater than 40, hospitalization in rural areas and small urban clusters and lack of health insurance as a predictor. Our analysis also revealed an increasing pattern of HRI hospitalizations over time. Our findings reinforce previous research on heat morbidity and explore the prevalence and use of air conditioning among vulnerable populations. Future research should employ spatial analytic techniques to identify environmental risk factors with focus on the differences between rural and urban heat-related morbidity. Additionally research should address common comorbidities associated with HRI and HRI's impact on hospital resource use and costs.
